# The research progress of ferroptosis in acute lung injury

**DOI:** 10.1016/j.bbrep.2025.102434

**Published:** 2026-01-21

**Authors:** Yixuan Bai, Yongming Ma, Xingfang Li

**Affiliations:** a College of Integrative Chinese and Western Medicine, Gansu University of Chinese Medicine, Lanzhou, China; b First Clinical Medical College, Gansu University of Chinese Medicine, Lanzhou, China; c Department of Respiratory and Critical Care Medicine, Gansu Provincial Traditional Chinese Medicine Hospital, Lanzhou, China

**Keywords:** Ferroptosis, Acute lung injury, Research progress

## Abstract

Ferroptosis, an iron-dependent form of regulated cell death driven by lipid peroxidation, is increasingly recognized as a pivotal mechanism in the pathogenesis of acute lung injury (ALI) and its severe form, acute respiratory distress syndrome (ARDS). Its core molecular machinery, including glutathione peroxidase 4 (GPX4), acyl-CoA synthetase long-chain family member 4 (ACSL4), and the cystine/glutamate antiporter system Xc-, becomes dysregulated across various ALI subtypes, such as sepsis, ischemia-reperfusion, and COVID-19.This review delineates how ferroptosis contributes to ALI through iron overload, uncontrolled lipid peroxidation, and failure of antioxidant defenses, ultimately leading to pulmonary endothelial and epithelial cell death. We further summarize subtype-specific mechanisms and evaluate emerging therapeutic strategies, including ferroptosis inhibitors (e.g., liproxstatin-1), Nrf2 activators, and iron chelators, highlighting their potential for targeted intervention in ALI/ARDS.

## Highlights

• Ferroptosis is driven by iron overload and lipid peroxidation in ALI.
• GPX4 inactivation and ACSL4 upregulation are core mechanisms.
• Targeting Nrf2 or using inhibitors (e.g., Lip-1) alleviates ferroptosis.
• It is a key pathway in sepsis, IRI, and COVID-19 related ALI.

## 1 Introduction

**Fig. 1:**
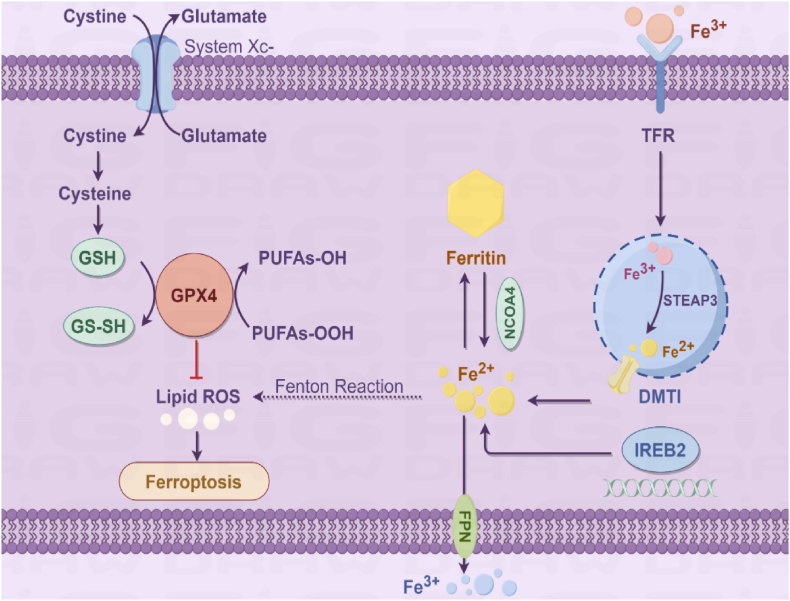
Schematic diagram of ferroptosis mechanism.

Acute lung injury/acute respiratory distress syndrome (ALI/ARDS) was first mentioned in 1915, when a Canadian soldier who inhaled toxic gas was recorded as having "shock lung" [1]. Later, in 1967, Ashbaugh et al. coined the term "acute respiratory distress syndrome" (ARDS), and its clinical symptoms were defined as acute dyspnea, hypoxemia, and loss of compliance following various stimuli. In 1994, the European and American Consensus Conference (AECC) released the definition of ALI/ARDS: radiologically, chest X-ray showed acute bilateral diffuse pulmonary infiltrates; an oxygenation index ≤300 was defined as ALI; and an oxygenation index ≤200 was defined as ARDS; pulmonary artery wedge pressure (PAWP) ≤18 or the absence of left atrial hypertension in clinical manifestations [2]. Acute lung injury (ALI) represents a severe inflammatory condition characterized by compromised integrity of both pulmonary endothelial and epithelial barriers [1]. The alveolar-capillary unit, comprising microvascular endothelium, interstitial space, and alveolar epithelium, becomes structurally and functionally impaired during ALI pathogenesis. Despite the application of standardized treatment modalities in clinical practice, the mortality rate of ARDS remains high, underscoring the urgent need to investigate novel pathological mechanisms and therapeutic targets. Key pathological hallmarks of this condition involve disruption of the alveolar-capillary barrier integrity, pronounced neutrophil infiltration into epithelial compartments, and elevated secretion of inflammatory mediators [3,4]. Biomarkers involved in inflammation and coagulation cascades found in both epithelial and endothelial cells can predict the incidence and mortality of ALI.The development of acute lung injury (ALI) is mediated through complex immune cell activation pathways. Multiple immune cell populations contribute to ALI progression, particularly alveolar macrophages, infiltrating neutrophils, adaptive lymphocytes, and circulating platelets [3,5]. In this context, ferroptosis, a novel form of regulated cell death, has recently garnered significant attention for its potential role in ALI pathogenesis, offering a fresh perspective for therapeutic development. These cellular components interact through intricate signaling networks to drive the inflammatory cascade characteristic of ALI. The pulmonary inflammatory cascade in ALI/ARDS involves coordinated cytokine production by resident (epithelial cells, fibroblasts) and recruited immune cells, creating a self-amplifying inflammatory milieu [6]. Currently, standardized treatment modalities are commonly used in clinical practice, including fluid resuscitation, broad-spectrum antibiotics, and basic life support. Despite significant improvements in disease prognosis due to optimized respiratory therapy, the mortality rate of ARDS remains as high as 25 %–40 % [7]. In recent years, the widespread outbreak of COVID-19 has led to around 15 %–20 % of patients developing severe cases, with approximately 5 % progressing to ARDS. The disease progression of acute lung injury/acute respiratory distress syndrome (ALI/ARDS) involves complex interplay between multiple programmed cell death pathways. These distinct yet interconnected mechanisms of cellular demise collectively contribute to worsening pulmonary pathophysiology through synergistic interactions. How to effectively treat ALI and ARDS remains a critical issue in respiratory and intensive care medicine, as well as public health，For a comparative overview of ferroptosis mechanisms across ALI subtypes, see Table 1, and a schematic diagram of ferroptosis pathways is provided in Fig. 1.

**Table 1:** Mechanisms and therapeutic strategies of ferroptosis in different types of acute lung injury.

Category	Sepsis-Associated ALI	COVID-19-Associated ALI	Universal Therapeutic Strategies
**Primary Trigger**	Bacterial LPS, systemic inflammation [8]	SARS-CoV-2 infection, hemoglobin-derived iron overload [9]	–
**Core Mechanisms**	• ACSL4/LOX-mediated lipid peroxidation [10,11] • GPX4 inactivation [8]	• Fe^2+^ overload (Fenton reaction) [9] TFRC1 upregulation [12,13]	Targets: • Iron metabolism (TFR1, ferritin) [14,15] • Lipid metabolism (ACSL4 inhibitors) [10,16] • Antioxidant system (GPX4 activators) [17,18]
**Key Pathways**	• TLR4/NF-κB pro-inflammatory pathway [8,18] • Nrf2/HO-1 antioxidant pathway [18]	• HIF-1α/HO-1 pathway (hypoxia) [19] • Nrf2 pathway suppression	Common pathways: • Nrf2-ARE activation [18,20]• LOX/ROS inhibition [16,21]
**Pathological Features**	Pericyte death, pulmonary edema, neutrophil infiltration [22]	Alveolar epithelial injury, hypoxemia, multi-organ iron deposition [9]	Shared hallmarks: • Mitochondrial damage [23,24] • Lipid ROS accumulation [25,26]
**Specific Therapies**	• Fer-1/liproxstatin-1[43,45] • Sevoflurane (antioxidant) [19] • Mesenchymal stem cells (BMSCs) [27]	• Iron chelators (deferoxamine) [15] • Lactoferrin (anti-inflammatory) [15] • Nrf2 activators (astaxanthin) [18]	Broad-spectrum inhibitors: • Liproxstatin-1[32] • Vitamin E [16] • Mitochondrial-targeted antioxidants (MitoQ)
**Clinical Challenges**	Poor in vivo stability of ferroptosis inhibitors [28]	Age/sex-dependent TFRC1 regulation [12,13]	Drug delivery efficiency and target specificity

Since its initial characterization by Dixon and colleagues in 2012, ferroptosis has emerged as a distinct iron-mediated regulated cell death pathway that has gained considerable research interest. This unique form of cell demise has been extensively investigated in multiple acute disease conditions, with growing evidence implicating its critical involvement in the pathogenesis of sepsis-induced acute lung injury. The ferroptotic process is fundamentally characterized by dysregulated iron homeostasis and excessive lipid peroxidation, manifesting as intracellular iron overload, toxic lipid peroxide accumulation, and subsequent plasma membrane destabilization leading to cellular demise [29]. Distinct from conventional cell death modalities (apoptosis, necrosis, or autophagy), the molecular signature of ferroptosis has positioned it as a prominent focus in contemporary biomedical research. This review aims to comprehensively examine current knowledge regarding ferroptosis in septic ALI, elucidate its pathophysiological contributions across different ALI subtypes, and evaluate emerging intervention strategies targeting this cell death pathway.

## 2 Basic mechanism of ferroptosis

Ferroptosis represents a distinct regulated cell death modality that differs fundamentally from classical apoptosis, necrosis, and autophagic cell death pathways [30]. The defining hallmarks of ferroptosis include its iron-dependent nature and characteristic lipid peroxidation cascade. The core molecular pathways involved in ferroptosis are summarized in Fig. 1.This unique cell death process is driven by iron-catalyzed peroxidation of polyunsaturated fatty acids within cellular membranes, ultimately resulting in catastrophic membrane damage and cellular collapse. The molecular pathogenesis of ferroptosis primarily involves three interconnected mechanisms: (1) dyshomeostasis of redox-active iron pools, (2) uncontrolled accumulation of phospholipid hydroperoxides, and (3) failure of glutathione-dependent antioxidant defenses.

(1) Abnormal Iron Ion Metabolism

Iron serves as an essential trace element that participates in numerous critical physiological functions, such as oxygen transport, mitochondrial respiration, and nucleic acid biosynthesis. Perturbations in iron metabolism, particularly pathological iron accumulation, represent a fundamental driver of ferroptosis [10]. Under physiological conditions, cellular iron exists in two redox states—ferric (Fe^3+^) and ferrous (Fe^2+^) iron—with their equilibrium meticulously maintained by sophisticated homeostatic mechanisms. This regulation encompasses the entire iron cycle, from intestinal absorption and systemic transport to cellular storage and elimination, with ferritin serving as the primary iron storage protein and transferrin acting as the key iron transport mediator.

During ferroptosis, dysregulated iron metabolism results in pathological iron accumulation, with preferential deposition of redox-active Fe^2+^. This labile iron pool catalyzes excessive hydroxyl radical (·OH) generation via Fenton chemistry, initiating a cascade of oxidative damage. The resultant oxidative stress induces widespread phospholipid peroxidation and subsequent cellular injury. Importantly, Fe^2+^ overload impairs physiological iron-regulatory feedback mechanisms, creating a vicious cycle that amplifies intracellular oxidative damage.

Emerging evidence indicates that ferroptosis is mechanistically linked to altered expression of key iron regulatory proteins, including hepcidin [31], divalent metal transporter-1 (DMT1), and ferritin. The iron-regulatory hormone hepcidin orchestrates cellular iron flux through modulation of iron transport systems, particularly by controlling transferrin receptor 1 (TFR1) activity and ferritin-mediated iron storage. Pathological ferroptosis frequently coincides with disrupted expression patterns of these iron homeostasis regulators, resulting in intracellular iron overload and consequent oxidative damage.

In physiological conditions, pulmonary iron homeostasis is precisely regulated through multiple integrated mechanisms: (1) ferritin-mediated iron sequestration, (2) antioxidant defenses in airway epithelia, (3) mucociliary clearance systems, and (4) alveolar macrophage phagocytic activity. Compromise of these protective systems leads to dysregulated iron metabolism, which can disrupt essential cellular processes. For instance, Wang et al. demonstrated that altered iron homeostasis may contribute to sepsis-associated acute lung injury pathogenesis, highlighting the therapeutic potential of targeting iron metabolism [32].These data indicate that pharmacologic regulation of iron homeostasis may therapeutically attenuate ferroptosis-mediated pulmonary damage.

(2) Accumulation of Lipid Peroxides

Lipid peroxidation represents the central biochemical cascade driving ferroptosis execution. The phospholipid bilayer, particularly its polyunsaturated fatty acid (PUFA)-containing species, serves as the principal target of oxidative attack during this process. The enzymatic process involves ACSL4-catalyzed activation of PUFAs, their esterification into membrane phospholipids by LPCAT3, and subsequent oxidation by lipoxygenases (LOXs) to generate cytotoxic phospholipid hydroperoxides.Notably, membrane susceptibility to peroxidative damage is governed by both the abundance and spatial organization of PUFAs within cellular membranes [25]. The ferroptotic peroxidation pathway initiates when ACSL4 (acyl-CoA synthetase long-chain family member 4) catalyzes PUFA activation to acyl-CoA derivatives. These activated fatty acids are subsequently incorporated into membrane phospholipids via LPCAT3 (lysophosphatidylcholine acyltransferase 3)-mediated remodeling, generating peroxidation-prone PUFA-phosphatidylethanolamines (PUFA-PEs) [10]. The final oxidative transformation involves LOX (lipoxygenase)-catalyzed conversion of these phospholipid substrates to cytotoxic phospholipid hydroperoxides (PLOOHs), which directly compromise membrane integrity [21].

Phospholipid hydroperoxides (PLOOHs), as terminal products of lipid peroxidation, serve dual biological functions in ferroptosis. Beyond serving as molecular signatures of ongoing ferroptosis, these oxidized lipids function as damage-associated molecular patterns that engage immune receptors—particularly Toll-like receptors (TLRs)—thereby amplifying inflammatory cascades and creating a feed-forward loop that exacerbates ferroptotic cell death [33]. This pathological interplay is particularly evident in acute lung injury/acute respiratory distress syndrome, where reactive oxygen species (ROS)-driven overproduction of PLOOHs establishes a vicious cycle of cellular damage and inflammatory exacerbation.

Current research demonstrates that PUFA-phosphatidylethanolamine (PUFA-PE) biosynthesis and membrane remodeling are critical determinants of both membrane structural integrity and cellular susceptibility to peroxidative damage [26]. These findings establish lipid peroxidation as a central mechanistic driver of ferroptosis, positioning its modulation as a key therapeutic target in ferroptosis-related pathologies. Notably, in ALI/ARDS pathogenesis, excessive reactive oxygen species (ROS) generation promotes elevated PLOOH formation. These oxidized phospholipids function as potent DAMPs that activate Toll-like receptor mediated signaling pathways, thereby amplifying the inflammatory cascade and tissue injury [11].

(3) Dysregulation of the Antioxidant System

The cellular antioxidant defense system is essential for maintaining redox homeostasis through two primary mechanisms: (1) enzymatic antioxidants including glutathione peroxidase 4 (GPX4) and catalase, and (2) non-enzymatic antioxidants such as reduced GSH and vitamin E. These systems work in concert to neutralize ROS and prevent oxidative damage. Ferroptosis pathogenesis is fundamentally linked to the collapse of these antioxidant defenses, with GPX4 dysfunction representing a critical molecular event in this cell death pathway. Specifically, GPX4 inactivation leads to uncontrolled lipid peroxidation through its inability to reduce phospholipid hydroperoxides, thereby triggering the ferroptotic cascade.

GPX4 serves as a pivotal antioxidant enzyme that maintains cellular redox homeostasis by catalyzing the glutathione (GSH)-dependent reduction of phospholipid hydroperoxides, thereby preventing their cytotoxic accumulation [17]. Mechanistic studies demonstrate that GPX4 inactivation or depletion results in uncontrolled lipid peroxide accumulation, which is both necessary and sufficient to initiate ferroptosis. The central role of the system Xc--GSH-GPX4 axis is further underscored by findings that its pharmacological inhibition robustly induces ferroptosis across multiple cell types. Furthermore, intracellular GSH concentration represents a key determinant of ferroptosis susceptibility [34]. GSH exerts its protective effects through iron chelation, limiting redox-active iron availability and subsequent Fenton chemistry. Under conditions of GSH depletion, liberated iron ions preferentially participate in Fenton reactions, generating deleterious hydroxyl radicals that amplify oxidative damage and accelerate ferroptotic cell death.

In addition to GPX4 and GSH, other antioxidant enzymes, such as SOD1 and SOD2, also play important roles in the process of ferroptosis. Studies have found that cells deficient in these antioxidant enzymes are more prone to ferroptosis under conditions of iron overload [35]. Therefore, dysfunction of the antioxidant system, particularly the reduction of GPX4 and GSH, becomes a key factor in the occurrence of ferroptosis.

(4) Other Mechanisms:

a. Exosomes and Ferroptosis

Exosomes are nanosized extracellular vesicles (30–150 nm in diameter) secreted by cells that carry diverse molecular cargo including proteins, lipids, and nucleic acids. These vesicles are ubiquitously distributed in biological fluids and have emerged as key mediators of intercellular communication. Recent advances reveal their significant involvement in regulated cell death pathways, particularly ferroptosis. Alveolar macrophage-derived exosomes have been shown to transport specific tRNA-derived fragments that activate HIPPO signaling and promote ferroptosis, a process that can be attenuated by ferroptosis inhibitors. Through their capacity to transfer bioactive molecules, exosomes modulate critical physiological processes including immune regulation, cellular motility, and differentiation [36].

Emerging evidence demonstrates that alveolar macrophage-derived exosomes transport specific tRNA-derived fragments (tRFs) capable of triggering ferroptosis via activation of the HIPPO signaling pathway. Importantly, pharmacological intervention with ferroptosis inhibitors (e.g., liproxstatin-1) significantly attenuates this cell death process, offering therapeutic potential for mitigating acute lung injury (ALI) pathogenesis [37].

b. Mitochondria and Ferroptosis

Mitochondria serve as the central bioenergetic hubs of eukaryotic cells, orchestrating diverse physiological processes including cell death regulation. A hallmark of ferroptosis is profound mitochondrial dysfunction, characterized by distinctive ultrastructural alterations: (1) organelle shrinkage, (2) cristae degeneration, (3) increased membrane density, and (4) outer membrane rupture [23].

Emerging research reveals that mitochondrial involvement in ferroptosis extends beyond ATP synthesis to include critical modulation of cellular redox homeostasis. Intracellular iron overload drives mitochondrial ROS overproduction, establishing a self-amplifying cycle of oxidative damage to both mitochondria and other cellular components. Notably, mitochondrial-targeted antioxidants (e.g., MitoQ) demonstrate therapeutic efficacy by scavenging mitochondrial ROS, thereby suppressing ferroptosis progression and mitigating acute lung injury (ALI) [24,38,39].

c. MUC1 and Ferroptosis

The transmembrane glycoprotein MUC1 mediates immunoregulation and maintains pulmonary barrier function, with clinical evidence supporting its role as a sepsis-to-ARDS progression biomarker [40]. Mechanistic studies demonstrate that disruption of MUC1 dimerization downregulates key antioxidant enzymes (including GPX4, GSH, and SOD) through the GSK3β/Keap1-Nrf2 signaling axis, while promoting lipid peroxide accumulation and mitochondrial membrane destabilization, ultimately triggering ferroptosis and aggravating pulmonary damage. This cascade is primarily mediated through the GSK3β/Keap1-Nrf2 signaling axis [32].

Experimental evidence reveals that bleomycin-induced lung injury is characterized by elevated lipid peroxidation markers (MDA, 4-HNE) and mitochondrial ROS accumulation. Intervention with mitochondrial-targeted antioxidants (e.g., MitoQ) effectively scavenges mitochondrial ROS, attenuates ferroptotic cell death, and ameliorates ALI [39,41]. These findings collectively highlight the dual roles of exosomes and mitochondria in maintaining cellular homeostasis and regulating ferroptosis pathways.

Ferroptosis pathogenesis involves a complex interplay of multiple intracellular pathways, including dysregulated antioxidant defenses, toxic lipid peroxide accumulation, and mitochondrial dysfunction. These findings position cellular organelles—particularly exosomes and mitochondria—as promising therapeutic targets for ferroptosis-associated pathologies such as acute lung injury and sepsis. However, current mechanistic understanding remains preliminary, and several critical questions must be addressed: (1) the precise spatiotemporal regulation of these organelles in ferroptosis, (2) their cell-type specific contributions, and (3) the feasibility of therapeutic targeting without disrupting essential physiological functions. Further preclinical validation and mechanistic studies are needed to translate these potential therapeutic strategies into clinical applications.

## 3 The role of ferroptosis in sepsis-related acute lung injury

Ferroptosis is an oxidative, iron-catalyzed cell death pathway characterized by phospholipid peroxidation under conditions of compromised cellular redox homeostasis.

Under homeostatic conditions, physiological levels of reactive oxygen species (ROS) function as crucial signaling molecules that regulate essential metabolic processes. However, redox imbalance—characterized by excessive ROS generation coupled with compromised antioxidant defenses—induces pathological oxidative stress. In the pulmonary microenvironment, this oxidative insult preferentially targets vulnerable cellular components, with lipid peroxidation-mediated membrane damage being particularly pronounced in both capillary endothelial cells and alveolar epithelial cells [42].

Iron overload represents a critical driver of oxidative stress through its catalytic role in free radical generation. Ferrous iron (Fe^2+^) catalyzes H_2_O_2_ decomposition via Fenton chemistry, generating cytotoxic hydroxyl radicals (·OH) that propagate oxidative chain reactions. These reactive oxygen species induce widespread cellular damage, while iron accumulation simultaneously potentiates lipid peroxidation cascades. The resultant membrane dysfunction and structural compromise ultimately trigger regulated cell death, thereby accelerating the pathogenesis of pulmonary conditions including ALI and ARDS [43].

Lipophilic compounds including CoQ10, α-tocopherol, and synthetic inhibitors (Fer-1, liproxstatin-1) suppress ferroptosis through distinct mechanisms: (1) CoQ10 regenerates reduced glutathione, (2) α-tocopherol terminates lipid peroxidation chains, and (3) Fer-1/liproxstatin-1 directly quench lipid radicals [16].

Nrf2 (Nuclear factor erythroid 2-related factor 2) serves as a master regulator of cellular redox homeostasis, functioning as a redox-sensitive transcription factor that orchestrates the antioxidant response. Under basal conditions, Nrf2 is sequestered in the cytoplasm through its interaction with the Keap1 (Kelch-like ECH-associated protein 1) repressor complex. Oxidative stress triggers conformational changes in Keap1, leading to Nrf2-Keap1 dissociation and subsequent Nrf2 nuclear translocation. Within the nucleus, Nrf2 binds to antioxidant response elements (AREs) in promoter regions, upregulating the expression of cytoprotective enzymes including superoxide dismutase (SOD), catalase (CAT), and glutathione peroxidase (GPx). This coordinated transcriptional program enhances cellular antioxidant defenses and protects against oxidative damage [18].

NF-κB, a master regulator of inflammation, coordinates innate and adaptive immune responses through transcriptional control of pro-inflammatory mediators. Current evidence indicates that NF-κB activation exerts inhibitory effects on the Nrf2 signaling pathway, creating a critical regulatory interplay between inflammation and oxidative stress. This antagonistic relationship significantly influences disease pathogenesis, where NF-κB-mediated upregulation of pro-inflammatory cytokines (TNF-α, IL-1β, and IL-6) amplifies inflammatory cascades, while Nrf2 activation confers cytoprotection through induction of antioxidant enzymes. The dynamic balance between these opposing pathways determines cellular fate under stress conditions [44,45].

In recent years, natural plant compounds, such as curcumin, ginseng, and astaxanthin, have been found to possess antioxidant and anti-inflammatory properties. These phytochemicals protect cells from oxidative stress and inflammation by scavenging excess ROS, enhancing the expression of antioxidant enzymes, and inhibiting the release of inflammatory factors. The antioxidant and anti-inflammatory properties of these natural substances provide strong support for their potential in treating diseases such as ALI/ARDS.

Accumulating evidence demonstrates that ferroptosis is mechanistically linked to various forms of lung injury, including ischemia-reperfusion-induced damage and sepsis-associated pulmonary dysfunction. Preclinical studies utilizing both in vivo models and in vitro systems have established ferroptosis as a key pathogenic mechanism in these conditions. Notably, the transcription factor Nrf2 has been identified as a master negative regulator of ferroptosis, orchestrating cellular defense against oxidative damage. Given its central role in mitigating ferroptotic cell death, therapeutic targeting of the Nrf2 pathway represents a promising strategy for attenuating oxidative stress and preserving pulmonary function in clinical settings.

(1) The Relationship Between Ischemia-Reperfusion Injury (IRI)-Induced Acute Lung Injury (ALI) and Ferroptosis

Intestinal ischemia-reperfusion injury (IIR) represents a critical medical emergency frequently encountered in clinical scenarios including intestinal volvulus, acute mesenteric ischemia, severe trauma, and circulatory shock. Importantly, acute lung injury (ALI) emerges as a predominant extraintestinal complication of IIR, establishing a critical organ cross-talk mechanism. The pathophysiology of IIR extends beyond local intestinal damage to systemic consequences, featuring: (1) intestinal barrier dysfunction, (2) microbial dysbiosis, (3) bacterial translocation, and (4) subsequent risk of sepsis and multi-organ failure. Notably, acute lung injury (ALI) emerges as a predominant extraintestinal complication of IIR, constituting a leading contributor to patient mortality [46].

Ferroptosis, an iron-dependent cell death process driven by lipid peroxidation and iron dysregulation, has been mechanistically implicated in intestinal ischemia-reperfusion (IIR)-induced mucosal injury [47]. Current evidence identifies three core pathological interactions: (1) HO-1-mediated iron overload, (2) GPX4 inactivation, and (3) ACSL4-dependent membrane phospholipid peroxidation.

Investigations employing intestinal ischemia-reperfusion (IIR) rodent systems consistently identify elevated expression of ferroptosis drivers COX2 and ACSL4, while exhibiting suppressed levels of the protective enzyme GPX4, collectively establishing a pro-ferroptotic molecular signature [47].These findings strongly implicate ferroptosis in IIR pathogenesis through two distinct yet complementary mechanisms: (1) COX2-mediated promotion of inflammatory lipid metabolism, and (2) ACSL4-facilitated generation of peroxidation-prone phospholipids. Specifically, COX2 drives the production of pro-inflammatory lipid mediators, while ACSL4 catalyzes the esterification of polyunsaturated fatty acids into membrane phospholipids, thereby providing substrates for lipid peroxidation - the biochemical hallmark of ferroptosis.

Nrf2 serves as a master transcriptional regulator of cellular redox homeostasis, mediating cytoprotective effects through upregulation of HO-1.However, under hypoxic conditions, HIF-1α-mediated overexpression of HO-1 may paradoxically exacerbate ferroptosis through excessive liberation of Fe^2+^ from heme catabolism, revealing the complex dual role of this pathway in IIR-ALI [20]. In IIR models, the Nrf2/HO-1 axis confers protection against oxidative damage and ferroptosis via two primary mechanisms: (1) degradation of pro-oxidant heme groups, and (2) subsequent reduction of labile Fe^2+^ pools. Paradoxically, hypoxia induces HIF-1α-mediated overexpression of HO-1, which despite its antioxidant properties, may exacerbate ferroptosis through excessive liberation of Fe^2+^ from heme catabolism (biliverdin/bilirubin pathway) [48]. Pharmacological intervention with glycyrrhizin demonstrates therapeutic potential by suppressing the HIF-1α/HO-1 axis, thereby reducing both lipid peroxidation and intracellular Fe^2+^ accumulation in pulmonary epithelial cells [19].

The Nrf2-STAT3 signaling axis plays a pivotal role in cellular stress responses. STAT3, a multifunctional transcription factor regulating immune/inflammatory processes and acute injury responses, is activated by Nrf2 and subsequently upregulates SLC7A11 expression. This cystine/glutamate antiporter (system xc-) component exerts anti-ferroptotic effects by: (1) enhancing glutathione synthesis and (2) mitigating iron overload, thereby protecting against IIR-induced tissue damage [49].

Furthermore, telomerase reverse transcriptase (TERT) emerges as a critical mediator of oxidative stress responses. TERT modulates cellular redox homeostasis by reducing ROS accumulation, thereby suppressing both oxidative stress and ferroptosis. Nrf2 maintains cellular integrity and mitochondrial function through TERT regulation, as evidenced by significantly diminished TERT expression in Nrf2-deficient models [50]. These findings collectively highlight an intricate regulatory network connecting Nrf2, STAT3, and TERT in ferroptosis modulation.

(2) Sepsis-Associated ALI and Ferroptosis

Sepsis is a systemic infection caused by pathogens such as bacteria and viruses, which triggers a widespread immune response and may lead to multiple organ dysfunction. Sepsis is often accompanied by diffuse alveolar and interstitial edema, causing severe hypoxemia and respiratory distress. In severe cases, it can progress to ARDS.

Pericytes, multifunctional perivascular cells essential for microvascular stability, play a pivotal role in sepsis pathophysiology. These specialized cells maintain vascular barrier function, regulate capillary tone, and participate in angiogenic processes. During sepsis, pericyte detachment and ferroptotic cell death contribute to: (1) microvascular barrier disruption, (2) increased pulmonary vascular permeability, and (3) subsequent acute lung injury (ALI). Experimental evidence demonstrates that ferroptosis inhibitors (e.g., ferrostatin-1) effectively preserve pericyte integrity and attenuate sepsis-induced ALI by inhibiting lipid peroxidation cascades [22].

Ferroptosis is an iron-dependent regulated cell death pathway characterized by excessive iron accumulation and lipid peroxidation. Emerging evidence strongly implicates ferroptosis in the pathogenesis of sepsis-associated acute lung injury (ALI). In Gram-negative bacterial infections, lipopolysaccharide (LPS) - the predominant outer membrane component - triggers a cascade of pathological events through TLR4-mediated innate immune activation. This LPS-TLR4 interaction induces: (1) profound pulmonary inflammation, (2) increased vascular permeability, (3) exacerbated pulmonary edema, and (4) ferroptotic cell death. Mechanistically, LPS promotes iron overload and lipid peroxidation, thereby establishing a vicious cycle of cellular damage and inflammatory exacerbation in the lung microenvironment [8].

Researchers have found that the ferroptosis inhibitor Fer-1 can suppress LPS-induced ferroptosis, reduce lipid peroxidation levels, alleviate pulmonary edema, and decrease lung vascular permeability. Fer-1 alleviates LPS-induced lung tissue damage by scavenging alkoxy radicals and inhibiting the downregulation of ferroptosis-related markers such as SLC7A11 and GPX4 [51].

While Fer-1 demonstrates significant anti-ferroptotic activity in vitro, its therapeutic efficacy in vivo is limited by pharmacokinetic challenges, including plasma instability and rapid metabolic clearance [28]. This limitation has prompted the development of next-generation inhibitors such as liproxstatin-1, which offers improved metabolic stability and maintains specificity for ferroptosis pathways independent of other cell death mechanisms. To overcome these limitations, next-generation ferroptosis inhibitors such as liproxstatin-1 have been developed, offering enhanced metabolic stability and specificity for ferroptosis pathways independent of other cell death mechanisms.

Beyond targeted inhibitors, the volatile anesthetic sevoflurane has emerged as a multifunctional therapeutic agent, exhibiting both anti-ferroptotic and anti-inflammatory properties. Mechanistic studies reveal that sevoflurane: (1) reduces labile Fe^2+^ pools and lipid peroxidation markers (e.g., malondialdehyde), (2) upregulates GPX4 expression, and (3) attenuates pulmonary inflammation by decreasing pro-inflammatory cytokines (IL-6, IL-1β, TNF-α) in bronchoalveolar lavage fluid. These coordinated actions collectively protect against ferroptosis-mediated lung injury.

Several natural plant-derived compounds have also been explored for their ability to inhibit ferroptosis. For instance, astaxanthin has been found to suppress LPS-induced ferroptosis by modulating the Keap1-Nrf2-HO-1 signaling pathway, thus reducing acute lung injury. Similarly, erythro-ascorbic acid (also known as methylene succinate or methylidene butanedioic acid) has been shown to activate the Nrf2 signaling pathway, decrease LPS-induced ferroptosis, and alleviate lung inflammation [18].

Moreover, stem cell therapy has been explored for its potential in mitigating sepsis-related acute lung injury. In a mouse model of lung injury induced by severe acute pancreatitis, transplantation of bone marrow-derived mesenchymal stem cells (BMSCs) effectively reduced iron deposition in lung tissue, alleviated lipid peroxidation, and improved lung injury [27], suggesting that stem cells can mitigate lung injury by modulating iron metabolism in addition to their known anti-inflammatory effects. This suggests that stem cells can not only suppress inflammatory responses but may also mitigate lung injury by improving iron metabolism.

These studies indicate that ferroptosis plays a critical role in sepsis-induced acute lung injury, and inhibiting ferroptosis can significantly reduce lung damage. Future research may further explore more stable and effective ferroptosis inhibitors and investigate the combined application of stem cell therapy, natural compounds, antioxidants, and other therapeutic strategies, providing new insights for the clinical treatment of sepsis-related acute lung injury.

(3) COVID-19-Related ALI and Ferroptosis

Acute Respiratory Distress Syndrome (ARDS) represents a frequent and life-threatening complication of severe COVID-19, primarily mediated through viral targeting of alveolar epithelial cells. This pathogenesis involves: (1) severe impairment of pulmonary gas exchange, (2) subsequent acute respiratory failure, and (3) a dysregulated immune response characterized by excessive cytokine and reactive oxygen species (ROS) production. This dysregulated immune response generates a cytokine-driven feedback loop that perpetuates both inflammatory and oxidative cascades, ultimately exacerbating lung injury in COVID-19-related ARDS.

SARS-CoV-2 infection not only affects alveolar epithelial cells but also causes the destruction of hemoglobin, leading to the release of Fe^2+^ into the bloodstream, resulting in iron overload. Excess Fe^2+^ in the bloodstream can aggravate oxidative stress, which is a significant mechanism of cellular damage. The surplus Fe^2+^ catalyzes reactions that generate harmful free radicals, further damaging cell membranes, proteins, and DNA [9].

Lactoferrin plays a regulatory role in immune responses and inflammation. By reducing the generation of cytokines and ROS, lactoferrin helps decrease oxidative stress and inflammation, thereby alleviating lung injury. Additionally, iron chelators can inhibit iron overload and prevent iron-induced oxidative damage, which may provide a potential therapeutic intervention [15].

Transferrin receptor 1 (TFRC1) is an important protein for the entry of iron ions into cells. Its expression is generally associated with factors such as age and sex. Studies have shown that the expression of TFRC1 is generally higher in males and elderly individuals compared to females, and this may be related to the higher mortality rate and the trend of disease progression in severe COVID-19 cases [12,13]. Although research in this area is ongoing, the specific role of TFRC1 in COVID-19 requires further clinical data for confirmation.

Due to the rapid mutation of the SARS-CoV-2 virus and the extensive multi-organ damage it causes, the complexity of diagnosis and treatment has significantly increased. Although vaccines and specific antiviral drugs (such as Paxlovid) provide some effective treatment options, clinical management, especially in the early stages, still primarily relies on broad-spectrum antibiotics, antiviral medications, corticosteroid treatment, and respiratory support. The significant role of iron metabolism and oxidative stress in COVID-19 infection, as well as the further cellular damage triggered by iron overload, are important areas of current research. Iron chelators and lactoferrin-based therapies are potential intervention strategies, but the clinical efficacy and safety of these approaches still require further investigation and validation.

## 4 Summary and outlook

Ferroptosis is an iron-dependent form of regulated cell death distinct from apoptosis, characterized by the iron-catalyzed accumulation of lipid peroxides. This review has systematically examined how the core mechanisms of ferroptosis—iron dyshomeostasis, lipid peroxidation, and antioxidant system failure—are engaged in a context-dependent manner across major ALI subtypes including sepsis, ischemia-reperfusion, and COVID-19. Emerging evidence has established its pathophysiological significance across multiple disease systems. In respiratory medicine, acute lung injury (ALI) and its progressive form, acute respiratory distress syndrome (ARDS), represent critical conditions where novel therapeutic strategies targeting ferroptosis could substantially improve clinical outcomes. Since its initial description in 2012, extensive preclinical research utilizing in vitro and in vivo models has systematically validated ferroptosis as a key mechanistic contributor to ARDS development. These studies have covered various pathological models, including ischemia-reperfusion-induced lung injury, sepsis-related lung injury, and lung injury following viral pneumonia. Researchers have explored the mechanisms by which different drugs affect specific signaling pathways and have proposed potential therapeutic strategies, aiming to provide more scientifically sound and effective interventions for the clinical treatment of ALI/ARDS. Looking forward, we propose three key research directions: (1) elucidating the precise regulatory nodes between inflammatory pathways and ferroptosis in specific lung cell types; (2) developing lung-specific ferroptosis inhibitors with improved drug delivery systems; and (3) validating ferroptosis-related biomarkers for patient stratification in clinical trials. Addressing these questions will facilitate the translation of promising preclinical findings into tangible clinical benefits.

Ferroptosis has firmly established itself as a key player in the complex pathophysiology of ALI/ARDS. The evidence reviewed herein supports the therapeutic potential of targeting this novel cell death pathway, either through direct inhibition or by modulating associated signaling networks. Future research focusing on the outlined directions promises to advance our understanding and ultimately improve outcomes for patients suffering from these devastating conditions.

## Informed consent

This study does not apply.

## Ethical approval

This study does not apply.

## Principle of integrity

Conducting research with integrity.

## Funding information

Gansu Provincial Natural Science Foundation Project, Grant/Award Number: 21JR11RA209.

## CRediT authorship contribution statement

**Yixuan Bai:** Writing – original draft, Writing – review & editing. **Yongming Ma:** Writing – original draft. **Xingfang Li:** Writing – review & editing.

## Declaration of competing interest

The authors declare that they have no known competing financial interests or personal relationships that could have appeared to influence the work reported in this paper.

## Data Availability

No data was used for the research described in the article.

## References

[1] Xu H., Sheng S., Luo W. (2023). Acute respiratory distress syndrome heterogeneity and the septic ARDS subgroup. Front. Immunol..

[2] Bernard G.R., Artigas A., Brigham K.L. (1994). The American-European Consensus Conference on ARDS. Definitions, mechanisms, relevant outcomes, and clinical trial Coordination. Am. J. Respir. Crit. Care Med..

[3] Bos L.D.J. (2022 Oct 1). Acute respiratory distress syndrome: causes, pathophysiology, and phenotypes.

[4] Matthay M.A., Zimmerman G.A. (2005). Acute lung injury and the acute respiratory distress syndrome. Centen. Rev..

[5] Butt Y., Kurdowska A., Allen T.C. (2016). A clinical and molecular review. Arch. Pathol. Lab Med..

[6] Reilly J.P., Calfee C.S., Christie J.D. (2019 Feb). Acute respiratory distress syndrome phenotypes.

[7] Tasaka S. (2022 Jul 8). Temporary removal: ARDS clinical practice guideline 2021.

[8] Li J. (2022).

[9] Habashi N.M., Camporota L., Gatto L.A. (2021). Functional pathophysiology of SARS-CoV-2-induced acute lung injury and clinical Implications. J. Appl. Physiol..

[10] Chen X., Li J., Kang R. (2021). Ferroptosis: machinery and regulation. Autophagy.

[11] Wu D., Spencer C.B., Ortoga L. (2024). Histone Lactylation-regulated METTL3 promotes ferroptosis via m6A-modification on ACSL4 in sepsis-associated lung injury. Redox Biol..

[12] McLaughlin K.-M., Bechtel M., Bojkova D. (2020).

[13] Borges Do Nascimento I.J., Cacic N., Abdulazeem H.M. (2020). Novel Coronavirus infection (COVID-19) in humans: a scoping review and meta-analysis. J. Clin. Med..

[14] Ru Q. (2024). Iron homeostasis and ferroptosis in human diseases: mechanisms and therapeutic prospects. Signal Transduct. Targeted Ther..

[15] Feng W., Xiao Y., Zhao C. (2022). New deferric amine compounds efficiently chelate excess iron to treat iron overload disorders and to prevent ferroptosis. Adv. Sci..

[16] Pope L.E., Dixon S.J. (2024).

[17] Stockwell B.R., Friedmann Angeli J.P., Bayir H. (2017). Ferroptosis: a regulated cell death nexus linking metabolism, redox biology, and disease. Cell.

[18] Luo L. (2022). Astaxanthin attenuates ferroptosis via Keap1-Nrf2/HO-1 signaling pathways in LPS-induced acute lung injury. Life Sci..

[19] Suresh M.V., Balijepalli S., Solanki S. (2023). Hypoxia-Inducible factor 1α and its role in lung injury: adaptive or maladaptive. Inflammation.

[20] Dong H., Qiang Z., Chai D. (2020 Jun 29). Nrf2 inhibits ferroptosis and protects against acute lung injury due to intestinal ischemia reperfusion via regulating SLC7A11 and HO-.

[21] Liang D., Feng Y., Zandkarimi F. (2023). Ferroptosis surveillance independent of GPX4 and differentially regulated by sex Hormones. Cell.

[22] Liu Y., Bao D., She H. (2024). Role of Hippo/ACSL4 axis in ferroptosis-induced pericyte loss and vascular dysfunction in Sepsis. Redox Biol..

[23] Gao Y., Mi N., Wu W. (2024). Transfer of inflammatory mitochondria via extracellular vesicles from M1 macrophages induces ferroptosis of pancreatic beta cells in acute Pancreatitis. J. Extracell. Vesicles.

[24] Liu Y., Lu S., Wu L. (2023). The diversified role of mitochondria in ferroptosis in cancer. Cell Death Dis..

[25] Pan Q., Luo Y., Xia Q. (2021). Ferroptosis and liver fibrosis. Int. J. Med. Sci..

[26] Qiu B., Zandkarimi F., Bezjian C.T. (2024). Phospholipids with two polyunsaturated fatty acyl tails promote Ferroptosis. Cell.

[27] Hu Y. (2022). Protection of Adipose-derived mesenchymal stromal cells during acute lung injury requires autophagy maintained by MTOR. Cell Death Discov..

[28] Li X., Duan L., Yuan S. (2019). Ferroptosis inhibitor alleviates radiation-induced lung fibrosis (RILF) via down-regulation of TGF-Β1. J. Inflamm..

[29] Dixon S.J. (2012 May 25). Ferroptosis: an iron-dependent form of nonapoptotic cell death.

[30] Feng S. (2023).

[31] Nemeth E., Ganz T. (2021). Hepcidin-ferroportin interaction controls systemic iron homeostasis. Int. J. Mol. Sci..

[32] Wang Y.-M., Gong F.-C., Qi X. (2022 Jul 21;2022). Mucin 1 inhibits ferroptosis and sensitizes vitamin E to alleviate sepsis-induced acute lung injury through GSK3β/Keap1-Nrf2- GPX4 pathway. Oxid. Med. Cell. Longev..

[33] Xue Q., Yan D., Chen X. (2023). Copper-dependent autophagic degradation of GPX4 drives ferroptosis. Autophagy.

[34] Kim J.W., Lee J.-Y., Oh M. (2023). An integrated view of lipid metabolism in ferroptosis revisited via lipidomic analysis. Exp. Mol. Med..

[35] Chen Y., Fang Z.-M., Yi X. (2023). The interaction between ferroptosis and inflammatory signaling pathways. Cell Death Dis..

[36] Wu W., Yu X., Wu J. (2021). Surface plasmon resonance Imaging-based biosensor for multiplex and ultrasensitive detection of NSCLC-associated exosomal miRNAs using DNA programmed heterostructure of Au-on-Ag. Biosens. Bioelectron..

[37] Wang W., Zhu L., Li H. (2022). Alveolar Macrophage-derived exosomal tRF-22-8BWS7K092 activates Hippo signaling pathway to induce ferroptosis in acute lung injury. Int. Immunopharmacol..

[38] Bersuker K., Hendricks J.M., Li Z. (2019). The CoQ oxidoreductase FSP1 acts parallel to GPX4 to inhibit Ferroptosis. Nature.

[39] Bock F.J. (2020 Feb). Mitochondria as multifaceted regulators of cell death.

[40] Tong X., Dong C., Liang S. (2024). Mucin1 as a potential molecule for cancer immunotherapy and targeted therapy. J. Cancer.

[41] Zhan P. (2022). Mitoquinone alleviates Bleomycin-induced acute lung injury via inhibiting mitochondrial ROS-dependent pulmonary epithelial Ferroptosis. Int. Immunopharmacol..

[42] Sies H. (2020 Jul). Reactive oxygen species (ROS) as pleiotropic physiological signalling agents.

[43] Fei Y., Huang X., Ning F. (2024). NETs induce ferroptosis of endothelial cells in LPS-ALI through SDC-1/HS and downstream pathways. Biomed. Pharmacother..

[44] Prescott J.A., Mitchell J.P., Cook S.J. (2021). Inhibitory feedback control of NF-κB signalling in health and disease. Biochem. J..

[45] Gu M., Jin J., Ren C. (2020). Akebia Saponin D suppresses inflammation in chondrocytes *via* the NRF2/HO-1/NF-κB axis and ameliorates osteoarthritis in Mice. Food Funct..

[46] Chu C., Wang X., Yang C. (2023). Neutrophil extracellular traps drive intestinal microvascular endothelial ferroptosis by impairing Fundc1-dependent Mitophagy. Redox Biol..

[47] Zhou H., Li D., Zhu P. (2018). Inhibitory effect of melatonin on necroptosis via repressing the Ripk3‐PGAM5‐CypD‐mPTP pathway attenuates cardiac microvascular ischemia–reperfusion Injury. J. Pineal Res..

[48] Xiong X., Lin Wang (2013). Sevoflurane attenuates pulmonary inflammation and Ventilator-induced lung injury by upregulation of HO-1 mRNA expression in Mice. Int. J. Nanomed..

[49] Qiang Z., Dong H., Xia Y. (2020 Sep 18). Nrf2 and STAT3 alleviates ferroptosis-mediated IIR-ALI by regulating SLC7A1. Oxid. Med. Cell. Longev..

[50] Dong H., Xia Y., Jin S. (2021). Nrf2 attenuates ferroptosis-mediated IIR-ALI by modulating TERT and SLC7A11. Cell Death Dis..

[51] Chen Z. (2023).

